# Clinical predictors of neurocognitive deficits in children with chronic kidney disease

**DOI:** 10.1007/s00467-006-0374-1

**Published:** 2007-04-01

**Authors:** Jennifer Slickers, Peter Duquette, Stephen Hooper, Debbie Gipson

**Affiliations:** 1 UNC Kidney Center, University of North Carolina, 7007D Burnett-Womack CB #7155, Chapel Hill, NC 27599-7155 USA; 2 Department of Psychiatry, University of North Carolina, Chapel Hill, NC USA; 3 Clinical Center for the Study of Development and Learning, University of North Carolina, Chapel Hill, NC USA

**Keywords:** Pediatrics, Chronic renal insufficiency, Developmental delay, Cognitive deficits

## Abstract

The purpose of the study was to explore associations between neurocognitive function and chronic kidney disease (CKD)-related clinical characteristics. Twenty-nine children, ages 7 to 19 years, with an estimated creatinine clearance (eCrCl) of 4–89 ml/min per 1.73 m^2^ body surface area were enrolled. Intellectual function (IQ), memory, and attention were measured and expressed as age-based standard scores. Clinical data were obtained by physical examination, laboratory testing, parental questionnaires and medical chart review. Pearson correlations and standard Student’s *t*-tests were used to identify significant (*P* < 0.05) relationships between targeted clinical variables and neurocognitive scores. Increased CKD severity correlated with lower IQ (*P* = 0.001) and memory function (*P* = 0.02). Memory function was lower in children with longer duration of disease (*P* = 0.03). Similarly, IQ scores were lowest when kidney disease had started at a younger age (*P* = 0.03) and with a greater percent of life with CKD (*P* = 0.04). Our findings provide preliminary evidence that increased disease severity, longer duration of disease, and younger age of onset of kidney disease potentially place children with CKD at increased risk of neurocognitive deficits. Additional investigation is required to better quantify these risk factors, particularly regarding how much variability is accounted for by these specific risk factors.

## Introduction

Neurocognitive difficulties have long been observed in the chronic kidney disease (CKD) population [1]. While identification and treatment of specific comorbidities of CKD (e.g., anemia) have yielded improvement in the overall functioning of this population, reports of neurocognitive deficits in children with CKD continue to appear. These neurocognitive deficits undoubtedly will have significant lifelong implications for the CKD population as they move into adulthood. A recent review by Groothoff [2] examined the long-term outcomes of children diagnosed with CKD and identified persistent difficulties with a limited knowledge base, memory, and concentration into adulthood, as well as lower educational attainment. A better understanding of potential risk factors for cognitive impairment and decline in the pediatric CKD population could allow for earlier intervention and, possibly, less morbidity, particularly for certain cognitive deficiencies.

Studies aimed at investigating neurocognitive impairment in children with CKD have identified a wide range of delays in motor and cognitive development. Measures of general intelligence, memory and attention have been used most frequently to look at cognitive function in the pediatric CKD population. Most studies have demonstrated lower IQ scores among children with end stage renal disease (ESRD) than in unaffected siblings [3] or the general population [4, 5] and also when pre- and post-transplantation performances are compared [6]. Memory deficits also have been identified in children with mild CKD as well as ESRD [7]. Fennell et al. [7] also reported in this same study that there was no significant decline in measures of attention among these children; however, improved attention performance has been demonstrated in children with ESRD after transplantation [6]. Observations of hyperactivity at school also have been noted in 50% of a study population assessing cognitive outcomes following dialysis during infancy [4]. The measures of cognitive performance in each of these studies varied widely but, when viewed collectively, suggest that children with CKD are vulnerable to cognitive deficiencies in IQ, memory and attention.

One thing to note, however, is that most studies to date have focused primarily on children with ESRD, leaving unanswered whether children with mild to moderate CKD also suffer this same vulnerability. Cognitive deficiencies in children with CKD could arise through a gradual process, proportional to the level of kidney dysfunction, or may develop once a filtration threshold has been passed. It seems logical that the severity of CKD might be proportional to the degree of cognitive impairment, but the data are not yet available to support this. The stage of neurologic development at the time of disease onset or the cumulative time children spend with CKD may also impact on the degree or type of cognitive impairment experienced. To our knowledge, no published study has yet focused specifically on isolating risk factors to identify which children with CKD are most at risk for cognitive decline. The primary goal of this study was to explore associations between clinical aspects describing CKD and selected neurocognitive test scores in a sample of children and adolescents with CKD. In addition, this study should provide the foundation for identifying specific clinical risk factors for subsequent neurodevelopmental dysfunction.

## Methods

### Participants

Study participants were recruited from a university-based pediatric nephrology practice. The participants included children between 7 and 19 years of age, with an estimated creatinine clearance (eCrCl) by Schwartz formula [8] of less than 90 ml/min per 1.73 m^2^ body surface area for at least 3 months duration. Renal transplant recipients and children with profound developmental delays were excluded. Following parental consent and patient assent, 29 children meeting these criteria underwent neurocognitive testing at a single point in time. A standardized physical examination and laboratory sample collection were also performed within 2 weeks of cognitive testing. A questionnaire providing demographic and historic information was completed by a parent or legal guardian. The study was reviewed and approved by the University of North Carolina Institutional Review Board.

Included among the study sample were 17 children with obstructive uropathy or structural congenital renal anomalies, seven with glomerulonephritis, one with calcineurin inhibitor toxicity, one who had sustained ischemic injury, one with renal insufficiency of undetermined etiology, one child with Alport’s syndrome, and one with cystinosis. Participants were evenly divided with respect to gender and disease severity. Approximately half the study sample had mild to moderate CKD with eCrCl ranging from 31–89 ml/min per 1.73 m^2^ body surface area. Kidney disease onset was documented from birth in 59%. The majority (59%) were hypertensive, and, among these children, 70% were on antihypertensive therapy, and 65% had measured blood pressure above the 95th percentile at the time of examination. The mean hemoglobin level was 12.7 mg/dl, with only three (10%) below 11 mg/dl, with values of 10.8 mg/dl, 10.7 mg/dl and 10.1 mg/dl. The general characteristics of our study sample are described in Table 1. All the participants attended school in a regular classroom setting, and two reported having an individualized education plan (IEP) at the time of participation.

**Table 1:** Demographic characteristics and medical variables of the study sample (*PD* peritoneal dialysis, *HD* hemodialysis)

Study sample characteristics	Number (*n* = 29)^a^	Range
Age (years)	12.5 (3.2)	7–19
Male (%)	52	
Caucasian (%)	52	
eCrCl (ml/min per 1.73 m^2^ body surface area)	32 (29)	4–89
ESRD (13 PD, 1 HD) (%)	48	
Age at disease onset (years)	4.4 (5.9)	0–16
Duration of CKD (years)	6.7 (4.7)	0.2–15
Percent of life with CKD	69 (39)	1–100
Hypertensive (%)	59	
Hemoglobin (mg/dl)	12.7 (1.5)	10–17

^a^Mean (standard deviation) or percent

### Neurocognitive instruments

All participants completed a battery of intellectual, attention, and memory tasks as part of a larger neuropsychological evaluation. To gain an estimate of overall intellectual functioning, we administered the Wechsler Abbreviated Scale of Intelligence [9]. The Gordon Diagnostic System [10], a computerized continuous performance test, was also administered to gain an estimate of attention functioning. Within the memory domain, the Wide Range Assessment of Memory and Learning [11] was employed to assess short-term memory across verbal and visual domains. Age-based standard scores (mean = 100, standard deviation = 15) were generated for variables from each of these tasks. In all instances, higher scores represented higher function for each cognitive test result. All tasks were administered by trained examiners who were supervised by a child neuropsychologist.

The Wechsler Abbreviated Scales of Intelligence (WASI) is built on the well-known Wechsler measures of global intellectual functioning [9]. It was designed for ages 6 years through adulthood, and employs a fluid-crystal model of intelligence. Four subtests comprise the WASI and include vocabulary, block design, similarities, and matrix reasoning. All four subtests were administered to gain a brief full scale IQ score.

The Gordon Diagnostic System (GDS) is a computerized continuous performance test that measures various aspects of visual attention [10]. For this study, the vigilance task was administered, which requires the individual to respond to specific visual stimuli (e.g., the number 1 followed by the number 9) from a series of numbers presented at a rate of approximately one per second. This task yields standard scores for the number correct, correct variability, commissions, and commission variability. For this study, the total correct standard score was employed.

The Wide Range Assessment of Memory and Learning (WRAML) is a comprehensive memory battery of tests [11]. It includes nine different subtests that measure short-term verbal memory (story memory, sentence memory, number/letter), short-term visual memory (picture memory, design memory, finger windows), and new learning capabilities (sound symbol, visual learning, verbal learning). A general memory index is also computed from the nine subtests. Normative data are available for ages 6 through 18 years. The WRAML was administered and scored according to standardized procedures, with the general memory index being employed for this study.

### Clinical measures

Disease severity, age of onset, and duration of renal disease, coded as time in years and as percent of life with CKD, as well as the presence of anemia or hypertension were explored as potential risk factors of interest, given their association with detrimental cognitive outcomes in the CKD literature. Clinical data were collected through physical examinations and laboratory tests performed within 2 weeks of cognitive testing. Age of disease onset, as well as duration and percent of life with CKD, were determined through record reviews and were based on the date of primary disease diagnosis.

The Schwartz formula [8], using creatinine and height measurements, was used to estimate the eCrCl for non-dialysis patients. Creatinine clearance and urea kinetics were calculated for peritoneal dialysis patients from a 24 h fluid collection. All but three of the peritoneal dialysis patients exceeded Kidney Disease Outcomes Quality Initiative (K/DOQI) guidelines for maintaining a Kt/V of at least 2.0, and those not achieving this level ranged from 1.8 to 1.9 [12]. For our continuous analysis, we represented the eCrCl from the 24 h fluid collection from dialysis patients (incorporating residual renal function for the six dialysis patients who still produced urine) on a continuum with the eCrCl from the Schwartz formula that had been calculated for CKD patients. The one hemodialysis participant had a Kt/V of 1.34 and received dialysis four times a week. For analysis, we imputed this participant’s eCrCl, designating the mean creatinine clearance value from anuric peritoneal dialysis participants. For the dichotomous analysis, “mild to moderate” disease was categorized as an eCrCl of 30 to 89 ml/min per 1.73 m^2^ body surface area, representing those patients with stage 2 or 3 CKD as defined by K/DOQI guidelines [12]. Those with an eCrCl of less than 30 ml/min per 1.73 m^2^ or those on dialysis, consistent with stage 4 or 5 CKD, were categorized as “severe CKD/ESRD”.

Anemia was defined as a hemoglobin level of less than 11 mg/dl, reflecting the normative ranges of hemoglobin for children aged 9 years and above and the K/DOQI guidelines that specify a target hemoglobin level of 11–12 mg/dl in CKD [13]. Hypertension was defined as either the regular use of antihypertensive medications or the presence of a blood pressure greater than the 95th percentile for age, height, and gender [14].

### Data analysis

Initial summary statistical analysis was used to describe the total study sample. This included both descriptive analysis and graphical examination of all relevant variables. To address the primary research question for this study, we used bivariate analysis comparing neurocognitive scores and the clinical characteristics of the study sample, using Pearson’s correlations for all continuous variables. Trend analysis based on linear regression was also used when continuous variables were compared graphically. For those variables that, by definition, were dichotomous (e.g., hypertension), Student’s *t*-tests were used to compare the mean neurocognitive scores between categories. For those clinical variables for which an argument could be made either to express continuously or to dichotomize on the basis of a normal range (e.g., hemoglobin), both correlations and *t*-tests were used.

## Results

An initial inspection of the neurocognitive test scores showed that the sample of children and adolescents with CKD was mildly variable, with most of the participants falling within the low average to average range on most tasks. For the full study sample, the mean IQ score was 91 (range 61 to 117, SD 16), and the mean memory score was 88 (range 65 to 128, SD 16), both falling within the low average to average range when compared with normative expectations. The mean attention score of 96 fell well within the average range but did show much more variability (range 37 to 120, SD 23).

### Disease severity

**Fig. 1:**
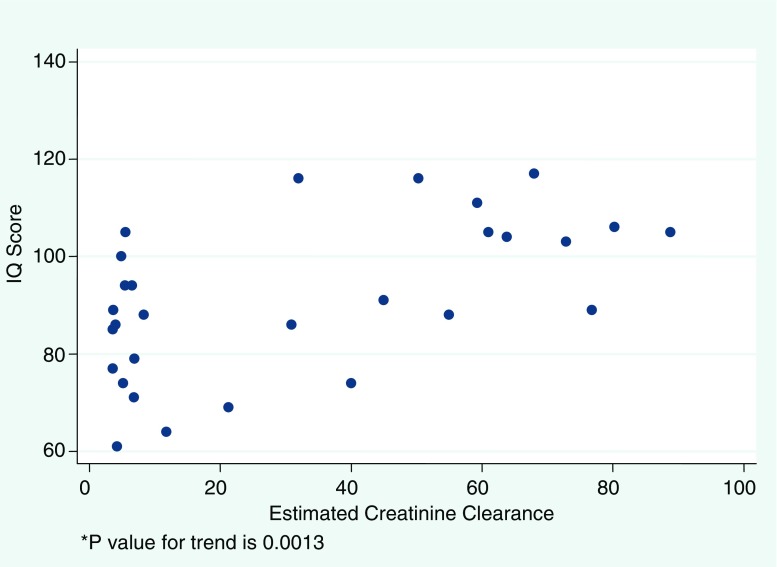
Relationship between IQ and estimated renal function

**Fig. 2:**
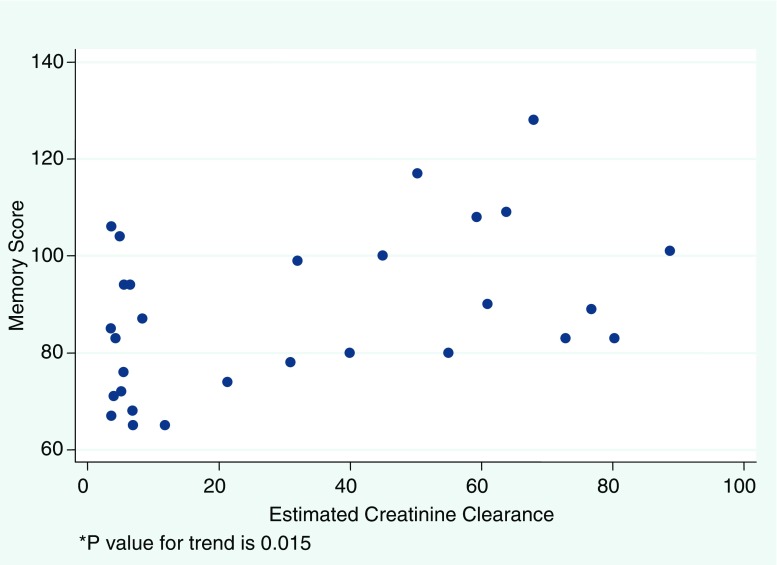
Relationship between memory and estimated renal function

**Fig. 3:**
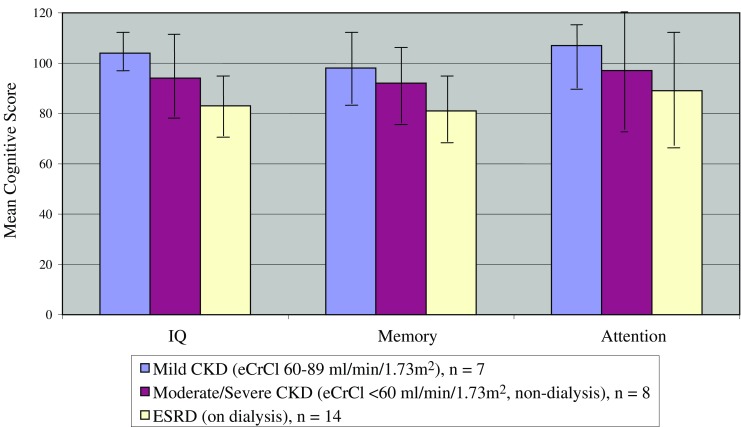
Mean cognitive scores by level of renal disease

Disease severity, represented by the eCrCl, yielded the strongest relationship with both IQ (*r* = 0.57, *P* = 0.001) and memory (*r* = 0.45, *P* = 0.02), demonstrating that higher eCrCl correlated with higher scores in these cognitive domains. Figures 1 and 2 illustrate the linear trend in the relationship between the continuous representation of disease severity and both IQ and memory scores. The correlation between attention and eCrCl was not significant. Additionally, lower IQ measures among severe CKD/ESRD participants in our study were evident when compared dichotomously with those with mild to moderate CKD (*P* = 0.0008), and the same relationships held for memory scores (*P* = 0.009) and attention scores (*P* = 0.012), despite the fact that attention was not linearly associated with eCrCl. Figure 3 illustrates the mean cognitive scores for categories of disease severity. While a downward trend with increasing severity is observed across all cognitive domains, statistical analysis confined to only the 15 non-dialysis patients yielded similar but less robust correlations among all areas of cognition, with IQ being the strongest (*r* = 0.46, *P* = 0.08).

### Age at disease onset and CKD duration

Increased CKD duration in years was associated with lower memory scores (*r* = −0.40, *P* = 0.03). Age at disease onset did not correlate significantly with IQ, memory, or attention scores in our original analysis. However, it was noted that many participants in our study sample had presented with already severe disease, making the date of disease diagnosis less reflective of the actual age at which reduced renal function began. In a secondary exploratory analysis, we repeated our analysis using only those subjects (*n* = 10) for whom we had a clearly documented date on which the eCrCl dropped below 90 ml/min per 1.73 m^2^. Among these patients were six with obstructive uropathy or structural congenital renal anomalies, three with glomerulonephritis, and one with cystinosis. In this secondary analysis we observed that the direction of prior relationships was preserved, with a stronger relationship between IQ and both age at disease onset (*r* = 0.69, *P* = 0.03) and percent of life with CKD (*r* = −0.66, *P* = 0.04). This may indicate that younger age at onset of CKD and longer percent of life with CKD correlate with lower IQ scores.

### Hypertension and anemia

Table 2 summarizes the results of our analysis of hypertension and anemia. The dichotomous separation of these two variables did not produce significant group differences. These findings suggest that the presence of anemia or hypertension at the time of testing did not correlate with differences in neurocognitive test results between study participants with or without these clinical features. In a secondary analysis we also explored designation of hypertension, defined initially as either the use of antihypertensive medications or the presence of hypertension at the time of examination, on the basis of the presence of each criterion separately, and we found no differences. Additional examination of anemia revealed that only three patients had a hemoglobin level below the anemia definition of < 11 mg/dl, with values of 10.8 mg/dl, 10.7 mg/dl and 10.1 mg/dl. Consequently, we were unable to assess anemic patients across a wide range of hemoglobin (Hb) values. Within our sample of hemoglobin ranging between 10.1 mg/dl and 17.0 mg/dl, no association between our cognitive domains and hemoglobin was observed.

**Table 2:** Relationships between hypertension, anemia and cognitive measures

Number (*n* = 29)	IQ^a^	General memory^a^	Attention^a^
Total cohort	91 (16)	88 (16)	96 (23)
Non-hypertensive	90 (18)	87 (16)	89 (24)
Hypertensive	92 (15)	89 (17)	100 (21)
Non-anemic	91 (16)	89 (16)	98 (22)
Anemic (<11 mg/dl)	92 (17)	81 (23)	76 (20)

^a^For each represented test among the normal population, the mean is 100 with a standard deviation of 15. No significant differences on Student’s *t*-tests for hypertension or anemia.

The use of a contemporary blood pressure or hemoglobin value to represent the hypertension or anemia status of our participants evaluated the potential effect of these factors on their cognitive performance on the day of testing. Day-to-day changes in blood pressure could cause changes in cognitive function. Another approach is to use time-averaged hemoglobin and blood pressure values as markers of long-term strain on cognitive skills. Therefore, we also did a retrospective chart review of all blood pressure and hemoglobin values recorded in the 6 months prior to the study visit and generated a mean value for each of these two variables. On the basis of these time-averaged values, we repeated our analysis. In the blood pressure range represented, hypertension remained unrelated to any of the cognitive variables. Hemoglobin levels, however, demonstrated a subtle correlation with memory performance in the continuous analysis (*r* = 0.41, *P* = 0.03). Dichotomous analysis of anemia yielded a significant difference in cognitive scores only when defined as a time-averaged hemoglobin level less than 10.5 mg/dl (mean memory score of 67 among anemic patients versus 91 among non-anemic participants, *P* = 0.01). However, this must be interpreted with caution, since only three participants fulfilled this criterion for anemia.

We should also acknowledge that there is a high degree of correlation among the results of the individual cognitive domains tested. Among the children included in our study, 12 (41%) had no cognitive scores greater than one standard deviation below the normative mean of 100, eight (28%) deviated in this fashion in a single cognitive domain, three (10%) did so in two areas, and six (21%) did so in all three areas. The level of overlap is, in part, likely due to the nature of cognitive testing, whereby testing in one area is, to some degree, dependent upon function in another area. The fact that there is not complete overlap, however, indicates that cognitive testing can distinguish among these deficits with some level of specificity.

## Discussion

Neurocognitive deficits among children with ESRD have been demonstrated in the literature, with some suggestion that key clinical variables can be associated with the level and, perhaps, the pattern of these deficits. The primary purpose of this study was to examine the relationship between targeted clinical variables, based on the available literature, and selected neurocognitive functions as defined by IQ, memory, and attention. Further, the current study examines these relationships across the full spectrum of CKD, including the mild to moderate stages of CKD. While it is recognized that some of the etiologies leading to CKD in our patients, namely cystinosis, calcineurin inhibitor toxicity, and ischemic injury, could potentially be independently associated with some level of cognitive dysfunction, in all three cases the children did not demonstrate evidence of other neurologic signs that would indicate central nervous system damage from their underlying disorders. Additionally, these children are representative of a typical pediatric CKD population, and any contribution from the presence of their comorbid conditions would not compromise the validity of our findings.

In the current study, several key findings were evident. First, disease severity significantly correlated with IQ and memory, while duration of disease significantly correlated with memory in the expected direction. Age at onset and percent of life with CKD did not initially correlate with any of the neurocognitive indices. When the age at onset and percent of life with the disease were examined in a secondary analysis restricted to a group of participants with more precisely defined onset of true decreased renal function, there was a clear trend that suggested that the younger the age at onset and the greater the percent of life with CKD, the lower the IQ score. The finding that younger age at renal insufficiency onset is significantly related to lower IQ complements previous observations in two prior studies of children with ESRD [3, 15]. Lawry et al. [15] found that among 24 children, including both dialysis and transplant patients, younger age at ESRD onset correlated with lower IQ scores. Brouhard et al. [3] also found that younger age at renal diagnosis was associated with lower achievement scores, although not specifically with lower IQ scores.

Also interesting to note is the wide range of IQ and memory scores among the ESRD subset of our study sample, as can be observed in Figs. 1 and 2. This might reflect the presence of other clinical variables, such as duration on dialysis, dialysis adequacy, or presence of residual renal function, which could also influence cognitive performance. A similar wide range of results among children with ESRD has been observed in other study samples, such as that of Brouhard et al. [3], in which children with renal disease had scores that included some in the average range but still had mean IQ and achievement scores significantly lower than their healthy sibling controls. These findings support the hypothesis that disease severity plays a strong role in predicting neurocognitive vulnerability, yet also suggest that there might be additional contributing variables characterizing end stage disease that warrant further investigation. In an effort to explore this issue further, the sub-analysis confined to the relationship between disease severity and cognitive outcomes in the non-dialysis participants demonstrated a more subtle downward trend of scores across all cognitive domains that did not reach statistical significance. This was likely, in part, due to the reduction in power resulting from our limiting the analysis to only 15 participants. It also might suggest that therapy with dialysis itself contributes to cognitive dysfunction.

The literature has been more ambiguous with regard to attention, and our findings did not demonstrate a significant deficit in attention. Our sample did, however, demonstrate significantly more variation in the attention measure among children with CKD than one would anticipate in the general population, as exhibited by the large standard deviation in our study sample. This suggests the possibility that there may be a subset of children with CKD who are more vulnerable to attention-related problems. More extensive exploration of the various components of attention may be required to explore this observation further.

Hypertension, as a single measure or time-averaged, was not associated with the neurocognitive outcomes evaluated. Hypertension in the general pediatric population has been correlated with decreased performance in tasks requiring memory, attention, and concentration [16]. Using school-aged children from a population-based US survey who were diagnosed with hypertension on the basis of systolic or diastolic blood pressure above the 90th percentile for age, height and gender, Lande et al. [16] showed that hypertension was significantly associated with lower neurocognitive scores representative of short-term memory, attention and concentration problems. In our study sample of children with CKD, the majority of our participants were on antihypertensive therapy, so any existing hypertension in our study sample was at least partially treated, thus significantly restricting the range of variance available for correlation analysis. Furthermore, some antihypertensive therapies, for instance clonidine, have the potential to cause sedating effects in children but can actually help concentration in other children. The effects of medications on just a few children could appear amplified in our small study sample, and, thus, a true relationship could be obscured.

The hemoglobin level at the time of testing did not predict cognitive function. Time-averaged hemoglobin concentrations less than 10.5 mg/dl were associated with lower memory performance. It has also been well established that anemia is associated with decreased cognitive function in adult patients with renal disease [17, 18]. Stivelman’s review on the topic highlights several studies investigating the use of erythropoietin in uremic adult patients. Increasing hematocrit was associated with improved neurocognitive measures [17]. For example, Marsh et al. [18] used erythropoietin in 24 adult hemodialysis patients to improve their average hematocrit from 23% to 36%, resulting in significantly improved neuropsychological test scores reflecting memory, learning and attention. In our study sample of children with CKD, only three children had a low hemoglobin level, and these were between 10.1 mg/dl and 10.8 mg/dl. The mean scores for memory and attention among those three children were 8 points and 22 points lower, respectively, than those for non-anemic children, suggesting that there could be a relationship that would require a larger sample size to identify. In the normal or near-normal range, detrimental effects of lower hemoglobin levels are likely more subtle and, thus, less likely to be captured in analysis of a small sample. Our time-averaged hemoglobin analysis supported this as well.

This is the first study to date that focuses specifically on identifying potential risk factors for neurocognitive decline in children and adolescents with CKD. Its value is further enhanced by the inclusion of children with mild to moderate CKD, thus extending findings to a wider range of pediatric CKD patients. Increased disease severity, duration of CKD, and younger age at CKD onset were identified as potential risk factors for the targeted neuropsychological functions of IQ and memory. Our results further suggest a significant linear relationship with disease severity, with IQ scores continuously declining as disease severity worsens. Not only does this finding argue against a threshold effect, it implicates disease severity as an important risk factor for neuropsychological dysfunction. These findings also suggest the possible utility of these variables in a cumulative risk model to predict neurocognitive dysfunction in CKD and lay the foundation for exploration of such a model. Finally, these findings emphasize the importance of conducting further research in neurocognitive development among children with CKD. This includes research both to more clearly predict which children with CKD are most vulnerable to neuropsychological dysfunction and to identify specific correlates of this dysfunction. While there have been great strides in improving quality and length of life among children with CKD, a better understanding of those processes that interfere with normal neurodevelopment is a critical element of providing optimal care to these children.
